# Radiometric Calibration for a Multispectral Sensor Onboard RISESAT Microsatellite Based on Lunar Observations

**DOI:** 10.3390/s21072429

**Published:** 2021-04-01

**Authors:** Masataka Imai, Junichi Kurihara, Toru Kouyama, Toshinori Kuwahara, Shinya Fujita, Yuji Sakamoto, Yuji Sato, Sei-Ichi Saitoh, Takafumi Hirata, Hirokazu Yamamoto, Yukihiro Takahashi

**Affiliations:** 1 Artificial Intelligence Research Center, National Institute of Advanced Industrial Science and Technology, Tokyo 135-0064, Japan; mstk-a.imai@aist.go.jp (M.I.); t.kouyama@aist.go.jp (T.K.); 2 AIST-UTokyo Advanced Operando-Measurement Technology Open Innovation Laboratory (OPERANDO-OIL), National Institute of Advanced Industrial Science and Technology (AIST), Kashiwa 277-0882, Japan; 3 Faculty of Science, Hokkaido University, Sapporo 001-0021, Japan; yukihiro@sci.hokudai.ac.jp; 4 Department of Aerospace Engineering, Tohoku University, Sendai 980-8579, Japan; toshinori.kuwahara.b3@tohoku.ac.jp (T.K.); shinya.fujita.a2@tohoku.ac.jp (S.F.); yuji.sakamoto.b4@tohoku.ac.jp (Y.S.); yuji.sato.s4@dc.tohoku.ac.jp (Y.S.); 5 Arctic Research Center, Hokkaido University, Sapporo 001-0021, Japan; ssaitoh@arc.hokudai.ac.jp (S.-I.S.); tahi@arc.hokudai.ac.jp (T.H.); 6 Geological Survey of Japan, National Institute of Advanced Industrial Science and Technology, Ibaraki 305-8567, Japan; hirokazu.yamamoto@aist.go.jp

**Keywords:** remote sensing, Earth observation, nano/microsatellite, lunar calibration

## Abstract

Radiometric calibration utilizing the Moon as a reference source is termed as lunar calibration. It is a useful method for evaluating the performance of optical sensors onboard satellites orbiting the Earth. Lunar calibration provides sufficient radiometric calibration opportunities without requiring any special equipment, and is suitable for nano/microsatellites. This study applies lunar calibration to a multispectral sensor, Ocean Observation Camera (OOC), on board a microsatellite named Rapid International Scientific Experiment Satellite. Simulating the brightness of the Moon based on the RObotic Lunar Observatory and SELENE/Spectrum Profiler models, sensitivity degradation was proven to be negligible in any of the four spectral bands of the OOC with the sensor temperature correction. A bluing trend in the OOC’s sensor sensitivity was revealed, indicating a shorter observation wavelength shows larger irradiance. Comparing the top-of-atmosphere reflectance of Railroad Valley Playa with the Radiometric Calibration Network dataset revealed that the derived calibration parameter from the lunar calibration was valid for correcting the bluing trend in the visible range. Although the lunar and vicarious calibration parameters for the infrared band were unexpectedly inconsistent, lunar calibration could potentially contribute toward estimating the contaminated background radiance in the Earth observation images.

## 1. Introduction

Earth observation by nano/microsatellites has been developing rapidly over the past decade [1]. The principal reason for this growing trend is the advantage of nano/microsatellites over large satellites in providing frequent observations of the Earth’s surface by satellite constellations. The PlanetScope constellation having the largest number of satellites in Earth observation with more than 130 nanosatellites in orbit, provides daily imagery of the entire Earth’s surface. In contrast, even a virtual constellation of the large Earth observation satellites composed of Landsat-8, Sentinel-2A, and Sentinel-2B provides an average revisit interval of 2.9 days [2]. The unprecedented high temporal resolution of the nanosatellite constellation opens new possibilities for Earth observation for the detection of short-term changes and acquisition of instantaneous information in case of illegal forest logging [3], water quality variations [4], and oil spill accidents [5].

However, in terms of radiometric precision, nano/microsatellite sensors still consist of many disadvantages arising predominantly due to size limitations and the use of commercial off-the-shelf components [6]. Significant radiometric inconsistencies exist between different satellites in the PlanetScope constellation [7]. Although several normalization methods have been developed for PlanetScope imagery using satellite images from Landsat-8, MODIS (Moderate Resolution Imaging Spectroradiometer), and Sentinel-2 [6,7,8,9,10], the acquisition time difference between the nano and large satellites erodes the advantage of the nanosatellite imagery with higher temporal resolution. In order to ensure radiometric precision, radiometric calibration is necessary for both nano/microsatellite as well as large satellite sensors, and thus, routine calibration and validation needs to be conducted. As nano/microsatellite sensors are relatively sensitive to launch vibration and space radiation environments, the on-orbit radiometric characteristics of the sensor may deviate from the pre-launch radiometric characteristics in both short-and long-time scales. Therefore, periodic, frequent on-orbit calibration for nano/microsatellite is very important to confirm variations in radiometric characteristics.

Onboard calibration hardware, such as solar diffuser panels and calibration lamp sources, generally employed for the on-orbit calibration of large satellite sensors [11], cannot be equipped on nano/microsatellites due to size limitations. Alternatively, vicarious and lunar calibrations can be applied for the on-orbit calibration of nano/microsatellite sensors. In case of vicarious calibration, sites such as dry lakes and deserts on the surface of the Earth, with known or measured ground-based reflectance values are chosen as calibration targets. The uncertainty of vicarious calibration depends on atmospheric measurements and cloud conditions at the site [12]. Inter-satellite observations with similar channels over Simultaneous Nadir Overpass (SNO) can also be used as a tool for calibration and relative stability monitoring. The uncertainty of cross-calibration with SNO approach depends mainly on radiometric precision of the reference satellite sensor. Lunar calibration uses the surface of the Moon as a calibration target, for which the reflectance was modeled from ground-based measurements and previous lunar missions. Although the reflectance of the Moon surface has the advantage of being highly stable (less than 1% variation over 1 billion years [13]) with no atmospheric effects, the actual implementation of lunar calibration is a challenging task for nano/microsatellites, which are controlled by limited attitude accuracy and power availability. While PlanetScope recently implemented a rigorous radiometric correction including lunar calibration [14,15], there are very few studies on the lunar calibration of nano/microsatellite sensors [16,17].

This paper demonstrates the results of lunar calibration applied to Ocean Observation Camera (OOC) on the Rapid International Scientific Experiment Satellite (RISESAT) microsatellite to establish a convenient and reliable radiometric calibration method for nano/microsatellite sensors. The objective of the OOC mission is to conduct ocean color remote sensing, which requires a relatively high radiometric precision among various fields of Earth observation. Compared with previous studies on the lunar calibration of nano/microsatellite sensors, the new approaches implemented in this study were (1) to conduct periodic and frequent Moon observations by the OOC for more than a year; (2) to use two different Moon models for radiometric calibration; and (3) to apply the sensor temperature of the OOC to the radiometric correction.

## 2. Materials and Methods

### 2.1. Satellite Sensor and Imagery

**Figure 1:**
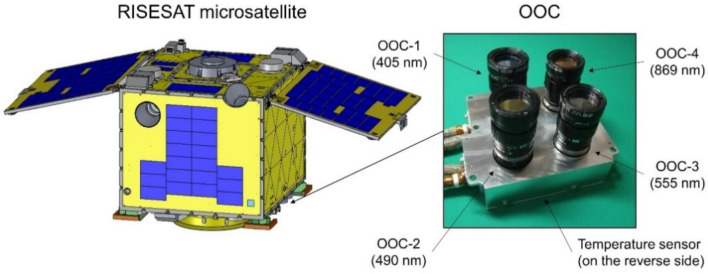
An overview of the RISESAT microsatellite and the Ocean Observation Camera (OOC) with its four cameras each corresponding to different spectral bands.

**Figure 2:**
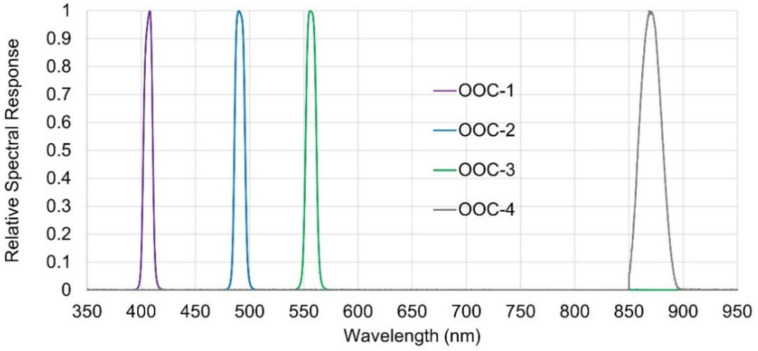
Relative spectral response functions of the OOC.

The OOC is one of the scientific instruments onboard the RISESAT microsatellite, which was launched on 18 January 2019, into a sun-synchronous orbit at an altitude of 500 km (Figure 1). The satellite bus system was developed by Tohoku University, and the scientific payload instruments were selected internationally from a wide variety of missions in Earth observation and space science [18,19]. The OOC was developed predominantly by Hokkaido University with funding and technical support from the National Taiwan Ocean University, PASCO Corporation, and Tohoku University. The objective of the OOC is to conduct ocean color remote sensing, which is focused on the colored dissolved organic matter (CDOM) in coastal waters. The OOC is a two-dimensional multispectral imager, with four cameras (OOC-1/2/3/4) corresponding to different spectral bands in the visible and near-infrared regions (Table 1). A bandwidth of approximately 10 nm (Figure 2) is typically required for ocean color sensors [20]. As an ocean color sensor, the OOC has a unique spectral band of OOC-1 at 405 nm dedicated for CDOM estimation. The spectral bands of OOC-2 and 3 are at 490 nm and 555 nm, respectively, and are conventionally applied for the estimation of chlorophyll-a concentration. In addition, the spectral band of OOC-4 at 869 nm is used for atmospheric correction. Each of the four cameras are composed of an optical bandpass filter (Andover Corporation, NH, USA), a lens with a fixed focal length of 50 mm (MORITEX Corporation, Saitama, Japan), and a charge-coupled device (CCD) image sensor (Watec Co., Ltd., Yamagata, Japan), which are commercially available at low costs. The four cameras are installed in a single housing with temperature sensor attached to the outer surface. In most large satellites, the sensor temperatures are precisely measured and controlled at each component. Contrarily, owing to the size and power limitations, the OOC contains a single temperature sensor in the housing with no active thermal control being performed.

Moon observations by the OOC started on August 16, 2019, after seven months of the initial launch checkout and preliminary observations for the scientific instruments onboard the RISESAT microsatellite. RISESAT was originally designed to track a ground target with an accuracy of 0.1° and 0.008°/s attitude stability. A high-speed attitude control computer and precision ground-tracking algorithms have been developed for this purpose [21,22,23]. This onboard attitude control system enabled the OOC to look at the Earth’s surface along with other astronomical objects including the Moon and other planets in our solar system. Table 2 provides a detail summary of the Moon observations by the OOC. The Moon surface brightness varies largely at a phase angle *|α|* ≤ 7°, as a strong backscattering or brightness opposition effect occurs at this value [24]. Thus, Moon observations were carried at an absolute phase angle of approximately 10° (± 2.5°) to obtain the maximum brightness, avoiding the backscattering surge. The observations were conducted every month until April 2020 and were repeated every 3–4 months afterward. The OOC acquired the Moon images with the four bands simultaneously, and multiple images were taken at intervals of a few seconds for each observation time.

### 2.2. Lunar Radiometric Models

Owing to the fact that the reflectance of the Moon’s surface is extremely stable, many studies have been conducted to utilize the Moon as a photometric standard. The major difficulty of using the Moon as an absolute radiometric standard exists in its strong brightness variations against solar illumination and viewing geometry. The U.S. Geological Survey in Flagstaff established the ground-based RObotic Lunar Observatory (ROLO) and developed a Moon model [25]. The ROLO model can predict the brightness of the Moon (irradiance) with a precision of ∼1% over a wide phase range. Using the ROLO model, inter-satellite calibration between Sea-WiFS and MODIS was demonstrated [26]. Currently the ROLO model and its implementation have been widely used in on-going satellites to detect the sensor degradation, such as Suomi National Polar-orbiting Partnership Visible Infrared Imaging Radiometer Suite, Landsat-8 Operational Land Imager, and so on [27,28].

Recently, another Moon model was developed using spacecraft observation data obtained by the Spectrum Profiler (SP) onboard the SELENE Japanese Moon orbiter [29,30]. The SP model can reproduce the Moon global brightness against any illumination and the viewing condition for any Moon location (including the opposite side of the Moon). This model has the advantage of calibrating an optical sensor with a higher spatial resolution and a field of view (FOV) narrower than the size of a disk. Although the absolute radiometric precision of the SP model (~10%) is large compared to that of the ROLO model, the measurement of relative sensor degradation of the order of 0.1% has been achieved [31].

**Figure 3:**
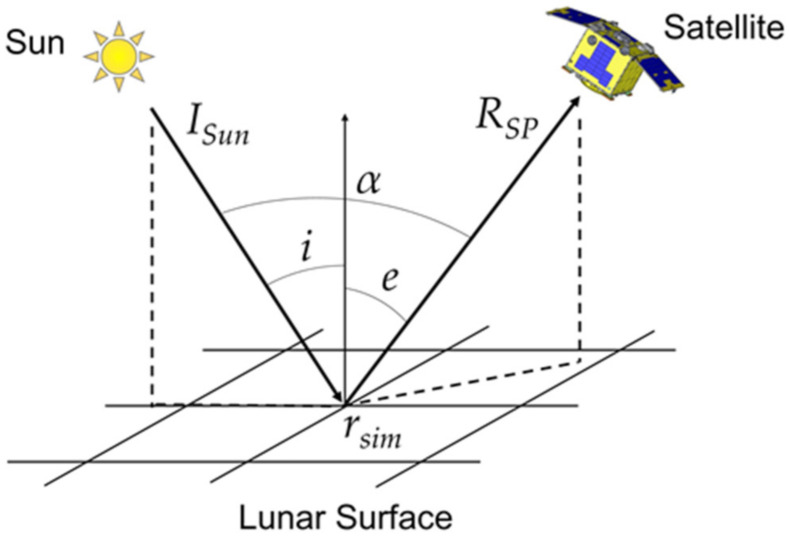
Definition of the solar incident angle (*i*), emission angle (*e*), and phase angle (*α*) used for the simulation of the lunar surface radiance (*R_SP_*) calculated from the solar irradiance (*I*_Sun_) and the radiance factor (*r_sim_*) in the Spectrum Profiler (SP) model.

In this study, both the ROLO and SP models were used to simulate the Moon irradiance received at the satellite position in the Earth orbit at each observation time. The SP model simulates the lunar surface radiance at each grid point specified by the solar incident angle (*i*), emission angle (*e*), and phase angle (*α*) (Figure 3). The photometrically corrected reflectance *r_corr_* is provided within the SP model, where *i* = 30°, *e* = 0°, and *α* = 30° is employed as a standard viewing geometry, and the radiance factor *r_sim_* is calculated as

(1) $$r_{sim}(\lambda ,i,e,\alpha )=r_{corr}(\lambda ,30^{\circ },0^{\circ },30^{\circ })\frac{X_{L}(i,e,\alpha )}{X_{L}(\lambda ,30^{\circ },0^{\circ },30^{\circ })}\frac{f(\alpha )}{f(30^{\circ })}$$

where, *X_L_* is a specific form of a disk function describing the *i*- and *e*- dependencies at a given *α*, and *f* is a phase function describing the *α* dependencies of the surface reflectance. The detailed forms of these functions are provided in the original paper [29]. In addition, the lunar surface radiance *R_SP_* [W m^−2^ μm^−1^ sr^−1^] is calculated as

(2) $$R_{SP}(\lambda )=r_{sim}(\lambda ,i,e,\alpha )\frac{I_{Sun}(\lambda )}{\pi }{\left(\frac{D}{1\mathrm{AU}}\right)}^{2}$$

where, *I*_Sun_ is the solar irradiance (W m^−2^ μm^−1^) at a distance of 1 AU, and *D* represents the distance between the Sun and the Moon in the astronomical unit (AU]. The positions of the Moon and the satellite were calculated using a two-line element set (TLE) of the satellite (www.space-track.org, accessed on 16 February 2021) and SPICE kernels [32]. The OOC’s relative spectral response (RSR) functions (Figure 2) were considered, and the disk-integrated Moon irradiance was predicted for each observation. It should be noted that the SP model covers only 512.6−1600 nm, whereas the ROLO model covers 350−2500 nm; thus, the shorter wavelength bands of OOC-1 and -2 were not compatible with the SP model.

**Figure 4:**
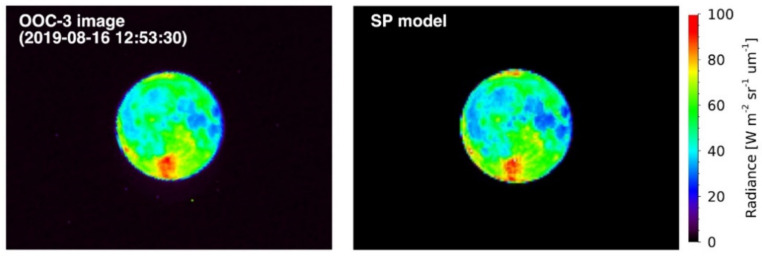
Moon images captured by the OOC−3 (555 nm) band (**left**) and simulated by the SP model (**right**). Both images are cropped to 165 × 124 pixels, and the observation image is rotated.

In the case of the SP model, the original output is a radiance map (Figure 4), which was converted to irradiance in the same manner as conducted for the observation image analysis (see Section 2.3). The SP model provides a radiance factor that corresponds to the reflectance standardized with the specified solar incident angle (*i*), emission angle (*e*), and phase angle (*α*) of 30°, 0°, and 30°, respectively. The disk-resolved lunar reflectance model has a resolution of 0.5° × 0.5° in lunar latitude and longitude based on SP hyperspectral data [30]. The SP model can generate the Moon image, with radiance values located at the center point of each pixel. As the effective spatial resolution of the SP model is better than that of OOC, the Moon image was simulated by the SP model with eight times higher resolution than OOC in advance. Later it was reduced to the same resolution by the binning process.

### 2.3. Radiometric Calibration

**Figure 5:**
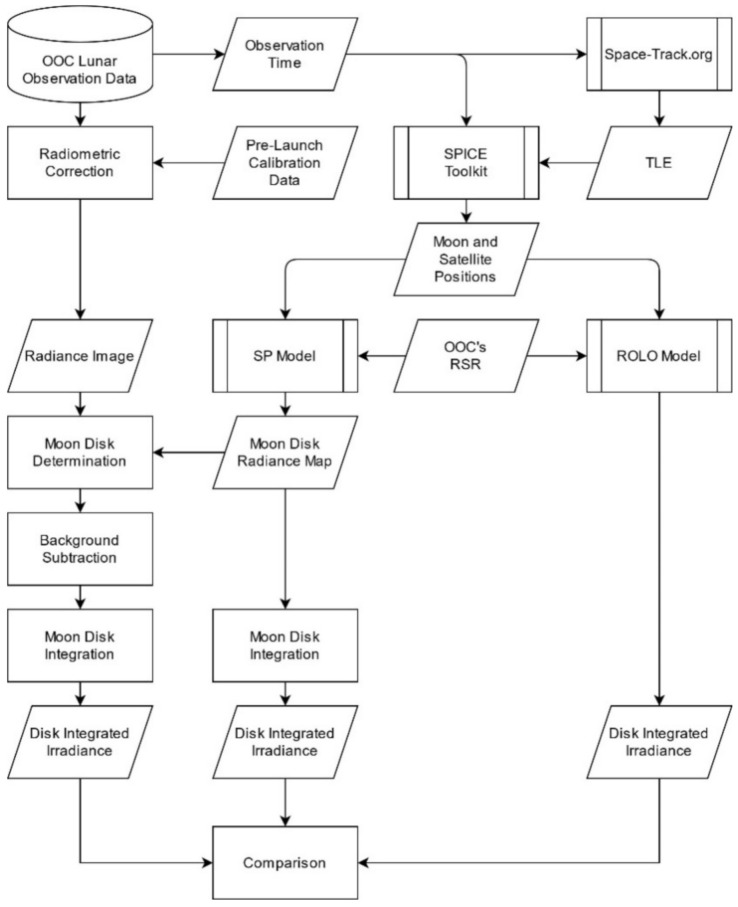
A flow chart of the lunar calibration using pre-launch calibration data.

**Figure 6:**
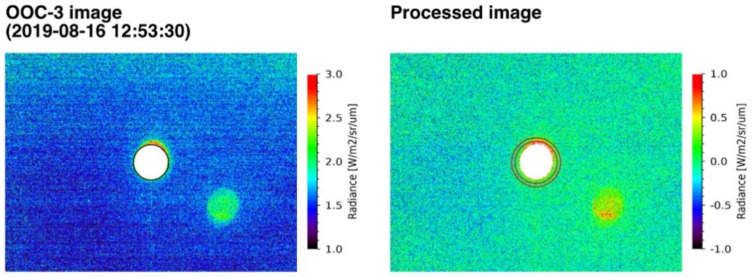
Low-illumination enhanced image of Figure 4 (**left**) and processed one after the subtraction of background radiance (**right**). Moon position was centered, and 500 × 375 pixels of surrounding background are displayed. Black circle in the left panel indicates the defined Moon limb (*L_P_* + 2 pixel). The additional background level was calculated from the red annular region shown in the right panel (see text for detail definitions).

Radiometric correction was performed for all the obtained OOC images using pre-launch calibration data (Figure 5). In advance of the radiometric correction, the observed Moon images were processed by bit conversion, offset reduction, and effective pixel extraction. The corrected radiance of each image pixel was stored as a 16-bit digital number, DN = *R_TOA_* (W m^−2^ sr^−1^ μm^−1^) × 100, where *R_TOA_* is the top-of-atmosphere (TOA) spectral radiance. Additional image correction was conducted to subtract background radiance. Figure 6 depicts the low-illumination enhanced image and processed image. To exclude the background, the center of the Moon in the pixel coordinates (*C_X_*, *C_Y_*) was identified by cross-correlation matching with the observation and SP simulated images. Considering the radius of the Moon *L_M_* [km], satellite–Moon distance *D_M_* [km], and the instrument instant FOV *θ_i_* = 1.483 × 10^−4^ (rad/pixel), the Moon disk area was defined as the inner region of Moon radius *L_P_* + 2 pixel, where *L_P_* = arctan (*L_M_*/*D_M_*)/*θ_i_*. The background radiance was measured in the annular region bounded by *L_P_* + 10 and *L_P_* + 20 circles, and the background was subtracted. In some images, ghost noise appeared in the off-Moon-disk area but may have resulted in a minor effect on the subtraction.

To compare with the irradiance predicted by the ROLO model, we need to integrate the irradiance (W m^−2^ μm^−1^) over the disk both for observation and the SP model-simulated images. The Moon irradiance was calculated as

(3) $$I=\sum_{i}R_{i}\omega$$

where, the subscript *i* indicates the *i*th pixel including the Moon disk region, *R_i_* is the radiance at the *i*th pixel, and *ω* is the instantaneous FOV of the pixels (in the case of OOC, *ω* = (1.483 × 10^−4^)^2^ (str)). The SP model tends to have a > 5% error in high incident-angle regions (near the terminator) and high-emission angle regions (near the limb). Kouyama et al. used the SP model excluding regions with solar incident angle *i* > 60° and emission angle *e* > 45° from their pixel-based comparison [30]. In this study, we integrated the entire Moon disk pixels because the small diameter of the observed Moon (~55 pixels) causes difficulties in excluding these regions. This point is discussed in Section 4 by comparing the results of the ROLO model.

## 3. Results

### 3.1. Sensor Performance Change and Temperature Dependences

**Figure 7:**
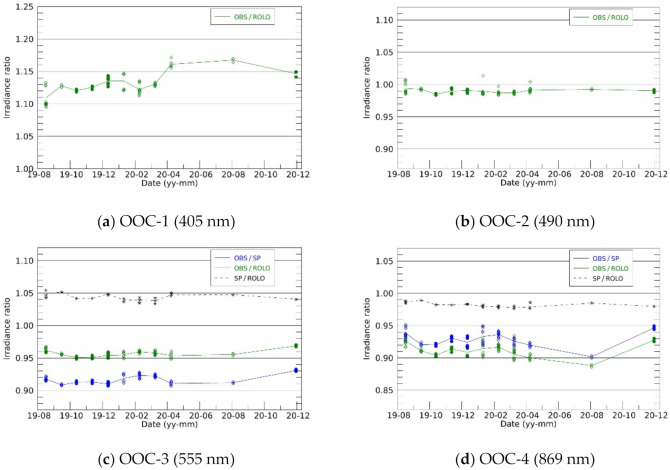
Temporal changes in the observation-to-simulation irradiance ratio (OSR) without the correction of temperature dependence of each sensor (see main text). The ROLO (green) and SP (blue) based OSR, and SP-to-ROLO irradiance ratio (black) are shown in OOC-3 and -4, where the SP model covers the observation wavelength. Waning Moon and waxing Moon are distinguished with filled circles and open diamonds, respectively.

The observed and model-simulated Moon irradiance were compared to investigate the sensor performance and its temporal change. Initially, the degradation of the sensors in space were evaluated. Figure 7 shows the temporal variations in the observation-to-simulation irradiance ratio (OSR). As the model could predict the Moon brightness precisely (~1% with ROLO), the sensor performance change could be identified as the relative change in the OSR. During 16 months of monitoring from August 2019, OOC-1 depicted an increasing trend in the OSR, while the other bands did not depict clear trends in the OSRs. The OSRs with the ROLO model changed by up to 3% in each observation month. The SP model irradiance was ~4% larger and ~1.5% smaller than the ROLO model irradiance in OOC-3 and OOC-4, respectively, while the deviation of the SP-to-ROLO irradiance ratio was <1%. The positive and negative lunar phase angles did not significantly affect OSR variation.

**Figure 8:**
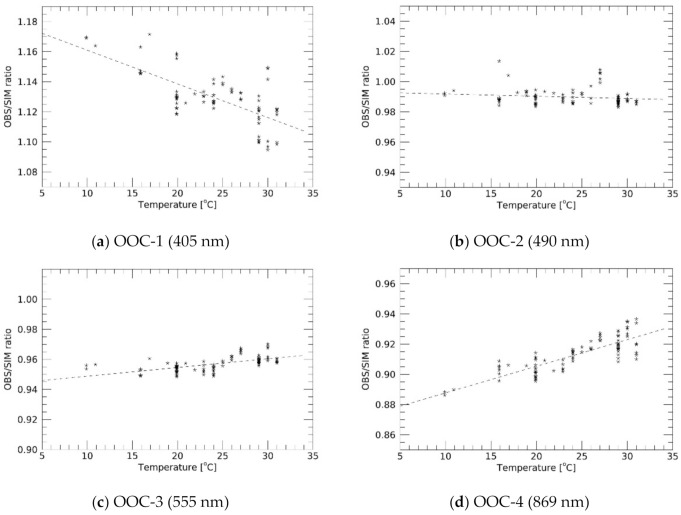
Observation-to-simulation irradiance ratio (OSR) scatters as a function of the sensor temperature. Dashed lines are the results of the linear fitting.

Our results presented a large variation in the OSRs, implying the existence of other factors affecting the sensor performance. In Figure 8, the sensor temperature was analyzed to determine the potential correlations with the OSRs. Although the OOC measured the sensor temperature not at the CCD image sensor but at the sensor housing (as indicated in Figure 1), it was found that the OSRs were dependent on the sensor temperature. The observed negative and positive dependences were major in OOC-1 and OOC-4, respectively, but they were minor in OOC-2 and OOC-3. Therefore, we performed linear fitting for each scatter and the derived slopes were −2.2 × 10^−3^, −1.4 × 10^−4^, 5.8 × 10^−4^, 1.8 × 10^−3^ (/°C) for OOC-1/2/3/4, respectively.

**Figure 9:**
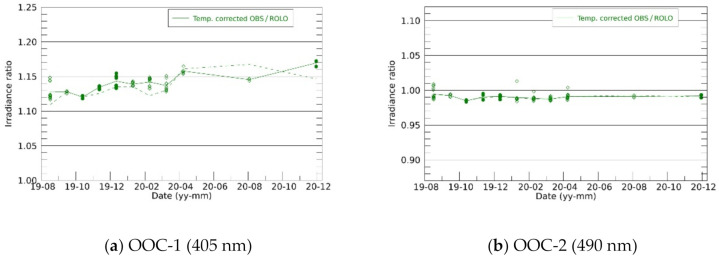

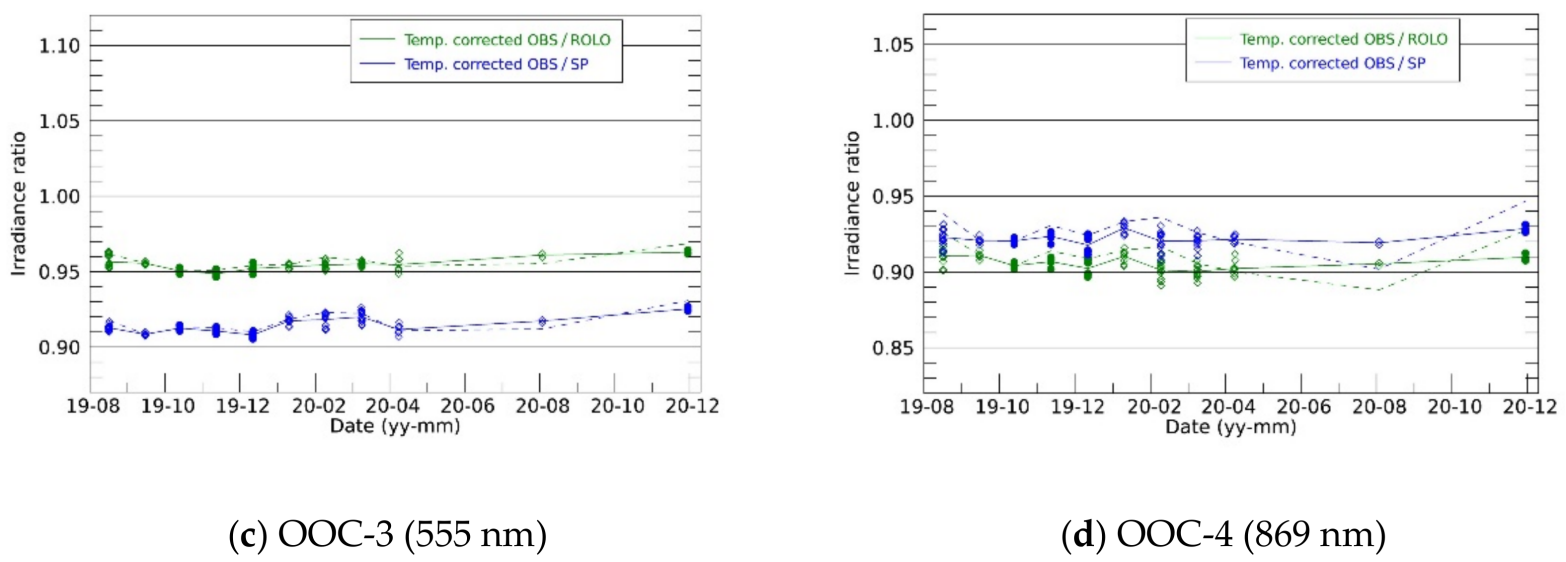
OSR plots from Figure 7, with corrected temperature dependences. Dashed lines indicate the OSRs before the correction.

Based on the linear fitting results, a temperature dependence correction was performed for all the OSRs to be normalized at a reference temperature of 20 °C. Figure 9 shows that the temperature-corrected OSRs do not exhibit obvious sensitivity degradation. OOC-1 still indicates an increasing trend; however, further observations are necessary to conclude this trend.

### 3.2. Validation of Lunar Calibration

**Figure 10:**
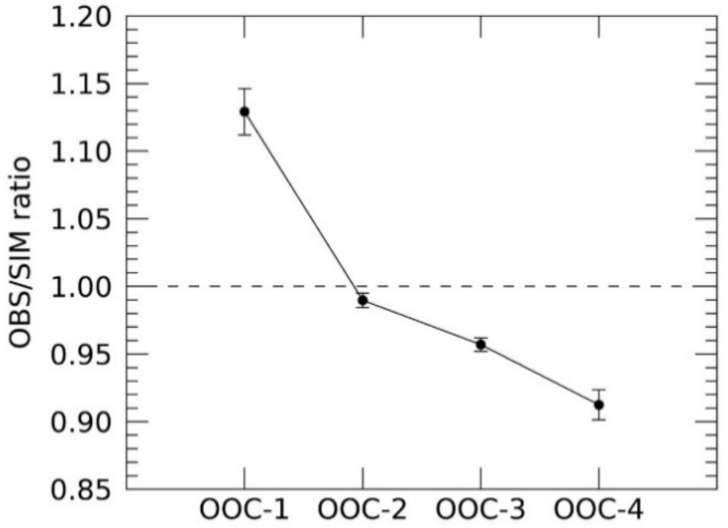
Averaged OSR for each observation band was obtained from all observation. Horizontal dashed line indicates the expected ratio based on the ROLO model, and the error bar indicates the standard deviation, in which the OOC’s temperature dependence is included.

A comparison between the observed and simulated irradiance could help to determine the discrepancy between the previous radiometric calibration based on the pre-launch experiment data, and the current lunar calibration. Figure 10 displays the averaged OSR against the ROLO model obtained from all the observations. There is a clear bluing trend in which OOC bands of shorter wavelengths indicate larger irradiance ratios. As the sensor sensitivity degradation cannot be confirmed in this study, the bluing trend might occur due to the launch impact or at the early stage of the satellite operation in space.

To validate this bluing trend, an observation of the vicarious calibration site was conducted. Validation of a result of sensor sensitivity calibration requires to confirm consistency of the result with other methods. One of the most reliable vicarious calibration methods is to observe a well-maintained calibration site in an appropriate condition with a simultaneous ground-based observation and weather monitoring. Five observations were attempted targeting Railroad Valley Playa, which is a vicarious calibration site located 150 km east of Tonopah, Nevada. Based on the site view image of Railroad Valley [33], an observation date of 20 October 2020 was selected as an appropriate date for validation when there were no clouds or cirrus clouds above the site. The radiometric calibration network (RadCalNet) TOA reflectance data, developed by a working group of the Committee on Earth Observation Satellites Working Group on Calibration and Validation (CEOS-WGCV) [34], was utilized. The radiometric calibration test site (RadCaTS) in Railroad Valley has a square area of 1 km × 1 km centered at a longitude of 115.690° W and latitude of 38.497° N. The OOC observed this area at 16:36:31.500 UTC with 10 × 10 pixels, when the local solar time was 09:09 and the viewing angle was 24.62°. The closest RadCaTS TOA reflectance data were recorded at 17:00 UTC; thus, there was a difference of ~30 min between the datasets. The TOA radiance of OOC (*R_TOA_*) was converted to the TOA reflectance as

*ρ* = *πR_TOA_d^2^/I* cos *Φ* (4)

where, *ρ* is the TOA reflectance, *d* is the Earth-Sun distance in astronomical units, *I* is the solar irradiance, and *Φ* is the solar zenith angle. The Chance/Kurucz solar irradiance spectral model from MODTLAN V5.2 was utilized, as it was used to predict TOA reflectance in RadCalNet [35,36,37]. The solar irradiance was calculated from the numerical convolution of the OOC’s RSR function with solar spectral irradiance, which is a merged spectrum of [38,39].

**Figure 11:**
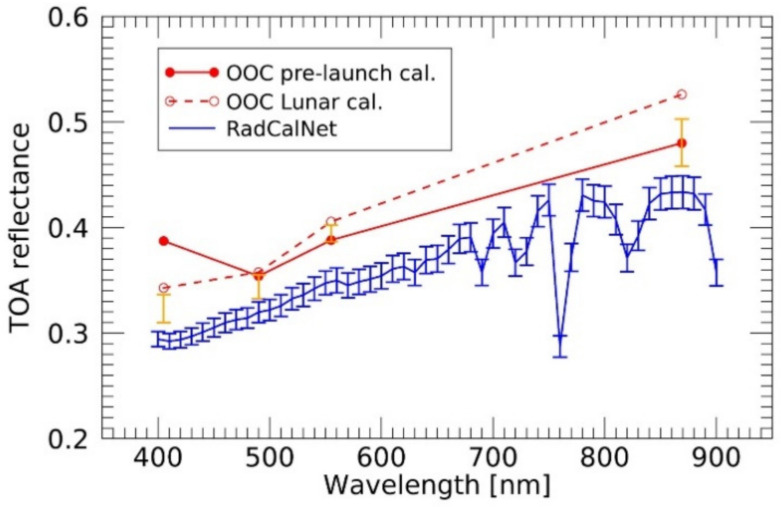
Top-of-atmosphere (TOA) reflectance spectrum of RadCaTS (blue) from RadCalNet and OOC observation (red). Both pre-launch calibration (solid line) and the current lunar calibration result (dashed line) are plotted, and the orange error bar indicates the potential spectrum change due to the superposed background illumination (see Section 4).

Figure 11 shows a comparison of TOA reflectance between the RadCaTS and OOC observations. After applying the lunar calibration result derived from Figure 10, the bluing trend recovered significantly and was consistent with the RadCalNet reflectance in the visible range. Although the observation time of the OOC was 30 min earlier than that of RadCalNet in the morning, the OOC’s TOA reflectance was slightly higher than that of RadCalNet. This might be attributed to the OOC observation viewing angle of 24.62°. Here, the uncertainties of the bidirectional reflectance distribution function effect and atmospheric correction was not considered. Although the lunar calibration provided reasonable corrections for OOC-1/2/3, the deviation in the OOC-4 was increased by lunar calibration. This deviation is discussed in detail in the following section.

## 4. Discussion

In this study, the two Moon models of ROLO and SP were utilized. The results of both models indicated that the OOC has no significant sensor sensitivity degradation. However, the SP model irradiance was ~4% larger and ~1.5% smaller than the ROLOs in OOC-3 and OOC-4, respectively. The original SP model has ~10% uncertainty in the absolute radiance, and there are correction coefficients to modify the SP radiance to be consistent with the ROLO’s integrated irradiance. The correction coefficients are a function of the wavelength, and the coefficients estimated by the previous work at a negative phase angle of −27.7° were used (see Section 3.2 of [30]). As our Moon observations were conducted in a rather small phase angle of 5.5°–12.5°, the discrepancy between the ROLO and SP model irradiance in OOC-3 and 4 was attributed mainly to the phase angle difference. Another possible explanation for this discrepancy is the relatively large uncertainty of the SP model radiance in the high-incident-angle region near the terminator of the Moon, and in the high-emission angle region near the limb of the Moon. Although the SP model has the advantage of providing disk-resolved Moon radiance, pixel-based comparisons were recommended solely in the regions where solar incident angle was less than 60° and emission angle was less than 45°, to avoid unexpected radiance bias [30]. However, the small discrepancy between the ROLO and SP models indicates that the SP model can be utilized to calculate the relative irradiance change even if the observed Moon image is small with a diameter of ~50 pixels.

Sensor sensitivity dependence on temperature is one of the main topics of this study. Although the OOC did not measure the sensor temperature precisely for each component, clear sensitivity dependencies on the sensor temperature were formed. The sensor temperature can be changed owing to the solar illumination conditions of the satellite in orbit and internal heating from the electric circuits. One of the notable results from this study is that the sensitivity dependence on temperature varies significantly with wavelength; a negative correlation was observed in OOC-1 at 405 nm, but positive in OOC-4 at 869 nm. Recently, a similar relationship was reported in optical navigation cameras (ONCs) onboard Hayabusa-2, which is a Japanese sample return mission from the asteroid’s surface (see Section 3.9.2 of [40]). Both studies indicate that shorter (longer) observation wavelengths have a negative (positive) correlation with temperature. In the OOC, the relationship is reversed at wavelengths ranging from 495 to 555 nm, but it was approximately 800 nm in the ONCs. Thermal analysis of the optical sensor system is required to elucidate this relationship. Although further interpretation is beyond the scope of this study, our results emphasize the importance of measuring the sensor temperature, especially for optical sensors in which commercial off-the-shelf components are used.

**Figure 12:**
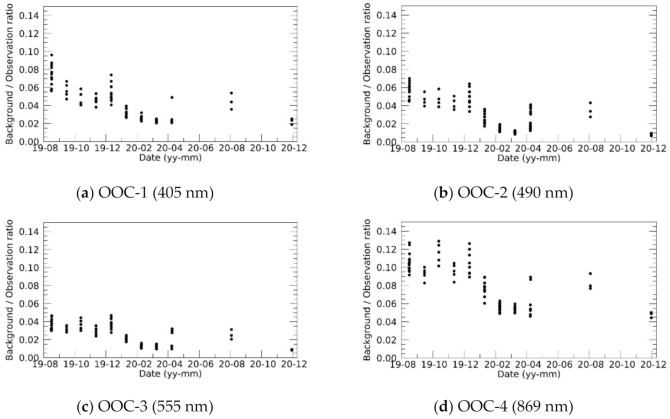
Temporal changes in the background-to-observation irradiance ratio.

After the correction of temperature dependencies, no significant sensitivity degradation was confirmed during the 16 months of the Moon observation period for half a year after the launch. However, a comparison of the observation and the ROLO’s simulation irradiance revealed a prominent bluing trend in OOC’s sensor sensitivity. This bluing trend was validated by comparing the TOA reflectance spectrum of the Railroad Valley Playa from RadCalNet. Although the derived re-calibration parameter is consistent for OOC-1/2/3, we could not have a conclusive interpretation of the deviation in OOC-4. One possibility is that the commercial lens used for OOC cameras is designed suitable for imaging in visible region, and thus OOC-4 band (infrared) is out of the design and may be contaminated by stray light in infrared region. Figure 12 depicts the background irradiance level, which can be caused by the contamination of stray light, to be subtracted from the Moon irradiance in the calibration process. From the analysis of Moon observations, the OOC-1/2/3/4 images have background contamination equal to ~4%, ~3%, ~2.5%, and ~8% of the Moon irradiance on average, respectively. The background contamination in OOC-4 is more than twice as large as that in the other bands; thus, similar background contamination will be included in the Earth observation images. In Figure 11, the possible reflectance spectrum is shown with orange error bars, assuming that background contamination was included in the images of Railroad Valley Playa. The minimum and maximum background-to-observation irradiance ratios were used to estimate the error bar range. Although the most appropriate cloud condition was selected from the five observations, the observation time and the viewing angle were not optimum for the validation. The different observation time may cause a disparity in atmospheric condition and the off-nadir viewing angle can contribute to bidirectional reflectance distribution function effects. Further vicarious observations are necessary to validate OOC’s bluing trend and to investigate the contamination in the Earth observation images, while the current results indicate that lunar observation could help to estimate the contamination of stray light in the Earth observation image.

## 5. Conclusions

Radiometric calibration using the ROLO and SP Moon models in space (lunar calibration) was conducted for a microsatellite named RISESAT launched in 2019 and its multispectral camera OOC. The OSR derived from both the ROLO and SP models can be utilized to reveal the dependence of the sensor sensitivity on the instrument temperature. After correcting the temperature dependency, no significant sensitivity degradation was observed in the OOC. Despite the small temporal change in sensitivity, there is a non-negligible discrepancy in the OOC’s sensor sensitivity, where a shorter observation wavelength shows larger irradiance against the ROLO model irradiance. To validate this bluing trend, vicarious observations targeting Railroad Valley Playa were conducted. By comparing the TOA reflectance obtained from RadCalNet, it was found that the derived parameter of the lunar calibration was tentatively valid for correcting the current OOC’s bluing trend in the visible range. Although the lunar calibration parameter for the infrared band was unexpectedly inconsistent with vicarious calibration, stray light contamination can be a plausible explanation, and lunar calibration could potentially contribute to estimation of the contaminated background irradiance in the Earth observation images.

Radiometric calibration with the Moon does not require any special equipment as long as the satellite satisfies the thermal balance and attitude control requirements for Moon observation. Therefore, lunar calibration can be a useful radiometric calibration method for optical sensors on nano/microsatellites, which have payload and cost restrictions. The relative irradiance derived from the SP model is consistent with that of the ROLO model with ~1% accuracy. As the SP model can provide a spatially resolved Moon radiance map, the SP model must be used for calibrating high-spatial-resolution optical sensors whose FOV is not wide enough to capture the full Moon disk.

## Figures and Tables

**Table 1:** Specifications of the OOC.

Size	388 × 161 × 124 mm
Weight	0.8 kg
Ground Sample Distance	74 m (at 500 km alt.)
Field of View	5.6° × 4.2° (48 × 36 km at 500 km alt.)
Spectral Bands	OOC-1: 405 nm OOC-2: 490 nm OOC-3: 555 nm OOC-4: 869 nm
Image Size	659 × 494 pixels
Data Quantization	10 bit

**Table 2:** Summary of the OOC’s Moon observations with phase angle and the sensor housing temperature listed for individual observation time. The number of obtained Moon images are indicated in the OOC band columns. Negative phase angles indicate a waxing Moon.

Observation Time	OOC Band				Phase	Sensor
	1	2	3	4	Angle (°)	Temperature [°C]
2019-08-16 05:00:52–05:01:08	4	5	5	5	−7.7	27.0
12:53:30–12:54:30	5	5	5	5	−11.1	29.0
16:02:30–16:03:30	5	5	5	5	−12.5	30.0
2019-09-14 23:56:31–23:57:31	5	5	5	5	11.0	19.9–20.9
2019-10-12 22:41:30–22:42:30	4	4	5	5	11.0	19.9
2019-11-11 16:01:30–16:02:30	5	4	5	5	10.7	24.0
2019-12-11 12:27:30–12:28:30	5	5	5	5	8.6	21.9–22.9
15:37:30–15:38:30	5	5	5	4	7.0	24.0–25.0
2020-01-09 21:51:30–21:52:30	4	5	5	5	−11.7	29.0
2020-01-10 01:00:30–01:01:30	5	5	5	4	−10.0	15.9
04:09:30	0	1	0	1	−8.5	15.9
2020-02-08 11:55:31–11:56:31	5	5	5	5	−12.0	29.0
15:04:45–15:05:30	4	4	4	4	−10.4	26.0
18:13:30–18:14:30	5	5	5	5	−8.9	31.0
2020-03-09 00:22:30–00:23:30	5	5	5	5	−12.1	19.9
03:31:30–03:32:30	4	5	4	5	−10.5	22.9
06:40:30–06:41:30	2	5	5	2	−9.1	22.9–24.0
2020-04-07 23:48:30–23:49:30	1	5	1	1	−6.0	15.9
2020-04-08 02:57:30–02:58:30	3	3	4	5	−5.5	19.9
13:57:30–13:58:00	1	2	1	1	−7.8	16.9
17:06:30–17:07:30	0	4	2	1	−9.2	18.9
2020-08-03 00:18:30–00:19:30	3	3	3	3	−8.4	9.9–10.9
2020-11-29 11:48:30–11:49:30	3	3	3	3	−9.6	30.0

## Data Availability

The data presented in this study are available on request from the corresponding author.
